# Editorial: Topic opportunities, barriers and pitfall of current nutritional practice in preterm infants

**DOI:** 10.3389/fped.2023.1172361

**Published:** 2023-04-05

**Authors:** Na Wang, Zhangbin Yu

**Affiliations:** ^1^ Department of Pediatrics, The Affiliated Suqian First People's Hospital of Nanjing Medical University, Suqian, China; ^2^ Division of Neonatology, Department of Pediatrics, Shenzhen People's Hospital (The Second Clinical Medical College, Jinan University), Shenzhen, China

**Keywords:** nutritional practice, preterm infants, colostrum, growth, human milk, amino acid profiles

**Editorial on the Research Topic** Topic opportunities, barriers and pitfall of current nutritional practice in preterm infants

In the past few decades, significant changes have been made to the nutritional practices for preterm infants. These changes mainly include people's increasing attention to the benefits of nutrient density on the health of preterm infants, immunobiology of human milk, and discussion of nutritional indicators.

Pulmonary edema is commonly observed in infants with bronchopulmonary dysplasia (BPD). However, while limiting fluid intake helps to prevent this condition, it can also limit energy intake (1). This highlights the importance of energy density for the health of preterm infants. Lin et al. conducted a cohort study with infants who had a gestational age of less than 28 weeks or a birth weight of less than 1,000 g, and they concluded that a higher proportion of enteral feeding/total fluid intake is associated with a lower risk of BPD. Therefore, preterm infants who do not have feeding intolerance should be encouraged to receive early and rapid enteral nutrition.

There are inevitably three issues regarding enteral feeding in preterm infants. The first was the initial time of enteral feeding, the second was the type of enteral feeding, and the third was the form of enteral feeding. Concerning the initial time of enteral feeding, Zhu et al. found that the first MOM feeding time in infants with very low birth weight (VLBW) >72 h after birth was a high-risk factor for moderate and severe BPD, suggesting that MOM feeding within 72 h of the birth of infants with VLBW reduces the risk of moderate and severe BPD. However, disputes remain regarding the type and form of enteral feeding (2). Human milk contains unique factors, including antibacterial, anti-inflammatory, immunomodulators, and living leukocytes (3). Human milk has many health benefits for preterm infants, including reduced incidence of necrotizing enterocolitis (NEC), late onset septicemia, BPD, retinopathy of preterm infants, and neurodevelopmental disorders (4). Therefore, breast milk is the first choice of enteral nutrition in preterm infants. The American Academy of Pediatrics has recommended exclusive breastfeeding for approximately six months, which has been universally recognized. At the end of 2019, COVID-19 broke out, hindering breastfeeding promotion in the neonatal intensive care unit (NICU). Jiang et al. conducted an education model based on WeChat procedures, ensuring that in public health emergencies, such as COVID-19 pandemic, the breastfeeding rate of high-risk infants who were vulnerable to infection would be unaffected during hospitalization in the NICU. Among preterm infants without human or donated milk, preterm infant milk powder or extensively hydrolyzed milk powder can be the choice of enteral feeding. Yin et al. demonstrated that in preterm infants less than 34 weeks, extensively hydrolyzed milk powder, compared with normal formula milk powder for preterm infants, can reduce feeding intolerance. However, it can also increase the incidence of metabolic bone disease within 6 months. Nevertheless, further research is required to determine whether extensively hydrolyzed milk powder can shorten the time of parenteral nutrition and reduce long-term complications. Significant differences exist in the method of nasogastric enteral feeding of preterm infants, especially in the continuous or bolus form and the frequency of feeding (5). The potential advantages and limitations of each form exist, and further research is required to confirm their advantages and disadvantages.

Colostrum is rich in nutrients and contains an appropriate proportion of sugar, fat, protein, and immune factors such as secretory immunoglobulin A (SIgA), lactoferrin, and lysozyme, which can regulate immunity, infection, and sterilization. However, clinical instability typically precludes enteral feeding in the first few days of life. Oropharyngeal administration is a potential alternative method for improving colostrum quality (6). To evaluate the effectiveness and safety of oropharyngeal administration of colostrum (OAC) in preterm infants, Huo et al. systematically evaluated the impact of OAC on preterm infants. OAC can reduce NEC incidence, late onset septicemia, and ventilator-associated pneumonia in preterm infants, shortening the time to reach full enteral feeding and the duration of hospital stay and increasing the rate of weight gain. Therefore, OAC can be used as part of routine care for preterm infants.

Currently, the nutritional indicators of NICU remain controversial. In the discussion of nutritional indicators, we no longer only seek to reduce the occurrence of extrauterine growth retardation (EUGR) because EUGR is a static process. At present, an increasing number of studies focus on dynamic EUGR, which refers to the change in body weight *Z*-score related to birth status. In 2021, a multicenter study from Jiangsu Province, China, found that the change in weight *Z*-score was closely related to the time to regain birth weight, birth weight, and gestational age (7). To reduce the growth failure of preterm infants after birth, sufficient attention should be paid to the time variable to regain birth weight in NICUs. In addition, a new concept of nutritional indicators has gradually approached NICU: the transition from parenteral to enteral nutrition. This phase indicated a gradual decrease in parenteral nutrition and a gradual increase in enteral nutrition. The duration of this phase is strongly associated with the growth failure of preterm infants at discharge (8). These are the two milestones of NICU nutrition indicators. To further improve the nutritional status of preterm infants, the transitional nutritional phase and its relationship with the disease status deserve further study.

This research topic on opportunities, barriers, and pitfalls of current nutritional practice in preterm infants presents the current progress in the nutritional practice of preterm infants. These studies have highlighted the importance of nutritional practices. However, in the field of preterm infant nutrition, there is still a long way to go in improving breastfeeding rates, effectively carrying out enteral nutrition, and reducing parenteral nutrition infusion time. Consequently, further discussion is required.
